# Assessing the FAIRness of survey data collected during the COVID-19 pandemic in Germany

**DOI:** 10.1371/journal.pone.0358755

**Published:** 2026-10-09

**Authors:** Tobias Gummer, Ko-Hsuan Lu, Karolina von Glasenapp

**Affiliations:** 1 GESIS Leibniz Institute for the Social Sciences, Data and Research on Society, Mannheim, Germany; 2 Heidelberg University, Max Weber Institute of Sociology, Heidelberg, Germany; Nanjing University, CHINA

## Abstract

This study investigates the FAIRness (Findability, Accessibility, Interoperability, and Reusability) of survey data collected during the COVID-19 pandemic in Germany. During the pandemic, surveys played a crucial role in capturing public attitudes and health-related behaviors, but researchers often faced a trade-off between rapid data collection and implementing high-quality survey designs. Using metadata from the “Survey Data Collection during the COVID-19 Pandemic” (SDCCP) project, which documents 717 surveys conducted in Germany, we assessed the FAIRness of surveys published with a persistent identifier or project website using the F-UJI assessment tool. Our results show substantial variation in FAIRness scores. Surveys can be divided into two groups, with one group achieving around 88.46% FAIRness and the other averaging around 65.38%. These differences are correlated with survey design. Surveys using fast and cost-efficient designs also exhibit lower FAIRness. The findings highlight that the trade-off between rapid data production and methodological rigor during the pandemic also affected the availability and reusability of survey data.

## Introduction

Surveys have been instrumental during the COVID-19 pandemic to capture attitudes and (health-related) behavior of the general public [1]. Based on these survey data, researchers were able to derive recommendations for political action [2,3]. Due to the high demand for rapid data collection at the onset of the pandemic, survey researchers in the social sciences were forced to make compromises in a trade-off between high-quality survey design and quick collection of data [1,4–6]. For instance, von Glasenapp et al. [7] and Gummer et al. [8,9] reported that surveys with design features often associated with lower data quality were more frequently used during the onset of the pandemic than high-quality designs. Especially, survey designs utilizing fast-to-implement web mode and less costly, quick-to-access non-probability online access panel samples were frequent during these times. These authors also showed that the survey projects that were quick to collect data and produce findings were less likely to publish data (quickly) and provide comprehensive documentation to facilitate re-use of their research data.

The availability and reusability of research data is key to the scientific process, whether for the purpose of replication or for the efficient use of research funding to produce data as a common good. As computer-assisted tools have become increasingly important for finding and using data, it is essential to enhance data management and stewardship so that it can be easily found, retrieved, integrated, and reused in future research [10–12]. To assess these features of datasets, Wilkinson et al. [12] proposed the FAIR principles that include Findability, Accessibility, Interoperability, and Reusability of research data. Findability means that the data can easily be found by both humans and machines. Accessibility indicates that the data can be further accessed and retrieved after being found. Interoperability indicates that the data is compatible with other data or tools. Reusability means that adequate information about the data is provided so that other researchers can reuse it. The FAIR principles can further be differentiated into 15 subdimensions and can be used as a framework for producing, documenting, and publishing data, as well as to describe and evaluate the FAIRness of existing data.

Previous research on the availability and reusability of survey data has either directly acknowledged and investigated the FAIRness of data or focused on specific aspects without directly making use of the FAIR framework (e.g., content of survey documentations). In the first category, focusing on election studies, Eder and Jedinger [13]reported that most election studies of Western democracies were well-documented and that their level of reusability was high. Adding to this, Zenk-Möltgen [14] showed that the FAIRness of national election data improved between 2018 and 2024. Albeit not only focusing on survey data, the studies by Batzdorfer et al. [15] and Sofi-Mahmudi et al. [16] argue that during the COVID-19 pandemic, the FAIRness of research data (esp. reusability) diminished.

In the second category, the studies by Jabkowski [17], Kołczyńska and Schoene [18], and Stefkovics et al. [19] investigate reporting standards in survey research. Jabkowski [17] focused on the reporting of key information on sampling, fieldwork procedures, outcome rates, and weighting. These pieces of information are central elements of survey documentation and reflect similar concepts as Reusability in FAIR (i.e., they enable data users to understand how data was collected and for which purposes they can be used). Closely related, Kołczyńska and Schoene [18] focused on documentation of sampling, outcome rates, questionnaire development, pretesting, and data quality controls. Adding a more nuanced view on outcome rates, Stefkovics et al. [19] investigated whether and how these were calculated and reported in journal articles using survey data. All three studies show that survey data documentation has improved in recent years. Focusing on the COVID-19 pandemic, von Glasenapp et al. [7] reported that during the onset of the pandemic, survey projects were more likely to focus on publishing first results quickly and provide less timely data access and thorough documentation. Further, these authors showed that data from survey projects that utilized easy and quick-to-implement survey designs (i.e., non-probability web surveys) were less accessible and interpretable compared to other survey projects. The authors concluded that this was the result of a trade-off within survey projects between quality and rapid data collection. Similar findings were reported by Gummer et al. [8], who showed that newly established longitudinal surveys during the COVID-19 pandemic focused on the quick production of results and less so on generating well-documented and accessible data.

Both lines of research have gaps that are detrimental to a better understanding of the FAIRness of survey data during the COVID-19 pandemic. Studies focusing on FAIRness have not specifically examined survey data collected during the COVID-19 pandemic [13–16]. Even when focusing on research data on the COVID-19 pandemic [15,16], it remains an open question how these generalize to social science surveys. Moreover, in this line of research, characteristics of the survey design have been neglected. The latter gap is unfortunate as decades of survey research have made a case that survey design can impact data quality. If FAIRness would differ across surveys with different designs, replication, and transparency would be biased towards those designs with higher FAIRness.

In contrast, studies focusing on surveys have neglected the full scope of the FAIR principles [7,8,17–19] and only a few focused on the COVID-19 pandemic [7,8]. This gap is rather important since previous studies reported that during the pandemic, the need for producing data quickly might have pushed researchers to make decisions that resulted in less well-documented data [7,8], possibly interrupting a positive trend in more comprehensive survey documentations [17–19]. Further, studies in this line of research mostly have a rather narrow perspective and focus on elements of the study or methodological documentation [7,8,17–19] or limit their perspective to the general availability of data [7,8]. As before, with the exception of the most recent studies [7,8], this line of research also largely neglects differences across survey designs. Again, this research gap is unfortunate as transparency and replication would be especially important for surveys, which, due to their design, raise questions regarding possible quality issues—possibly introducing biased replication and accessibility potentials. To summarize, in previous research, we see research gaps with respect to the intersection of (i) investigating the full scope of the FAIR principles, (ii) considering the important time during the COVID-19 pandemic, and (iii) acknowledging differences in FAIRness across survey design.

With the present study, we aim to address the gaps in prior research for surveys collected during the COVID-19 pandemic in Germany. We selected Germany as a case because it is characterized by an advanced research landscape with well-developed research infrastructures. Among researchers, a general knowledge about the need for replicability and transparency can be expected, and infrastructures for research data management exist. Moreover, focusing on surveys conducted in the same country allows us to keep the country context of each survey comparable. Consequently, we address the following research questions (RQs) on the FAIRness of survey data collected during the COVID-19 pandemic in Germany:

RQ1. How FAIR are survey data collected during the COVID-19 pandemic in Germany?

RQ2. Does the FAIRness of survey data collected during the COVID-19 pandemic in Germany differ by survey design?

To answer our research questions, we utilized data collected as part of the “Survey Data Collection during the COVID-19 Pandemic” (SDCCP) project [20] that includes metadata on 717 surveys collected during the COVID-19 pandemic in Germany [21]. We assessed FAIRness scores for those surveys published under a persistent identifier (i.e., DOI) or a project website utilizing the F-UJI assessment tool [22,23].

In the next section, we detail our data and methods. After presenting our results, we offer concluding remarks and outline opportunities for future research.

## Data and methods

### Data

The SDCCP project collected metadata on social science and public health surveys fielded in Germany between March 1st, 2020 and December 31st, 2021 [20]. The project covers surveys that have defined the German general population as the target population (i.e., which can be considered general population surveys). Metadata collected by the SDCCP project includes information about key survey design decisions (e.g., sampling strategy and mode choice) and data availability (e.g., DOI, project website, documentation content). All information was manually coded by experts and reached excellent intercoder reliability [20].

Surveys were selected for inclusion in the SDCCP dataset in a systematic multi-stage process [20]. First, using data archives, lists of surveys, and manual searches, a list of surveys was compiled. Second, the preliminary list of surveys was screened based on a list of eligibility criteria (e.g., target population, availability of basic information about the survey).

To answer our RQs, we relied on the SDCCP1 dataset release v1.0.0 [21]. SDCCP1 includes information on surveys that targeted the German general population and covers 717 surveys from 183 survey programs.

### Operationalization

We utilized the F-UJI assessment tool version 3.5.1 to systematically and automatically evaluate the FAIRness of each survey in our dataset [22,23]. F-UJI uses 17 testable metrics corresponding to the 15 subdimensions of FAIR to generate an overall FAIRness score, as well as individual scores for Findability, Accessibility, Interoperability, and Reusability, respectively [12,22]. These FAIRness scores describe how many of the FAIRsFAIR Data Object Assessment Metrics are fulfilled by a dataset. The number of metrics varies slightly between subdimensions, with Findability and Accessibility having a total of 7 metrics, whereas Interoperability and Reusability have 6 metrics. For example, one metric for Findability is “Metadata includes descriptive core elements to support data findability”, whereas “Data is accessible through a standardized communication protocol” is a metric of Accessibility. For technical descriptions and details for how F-UJI processes queries, we refer readers to the technical documentation and description of the tool [22,23].

We inputted the persistent identifier of each survey in F-UJI and obtained scores for each subdimension as well as an overall FAIRness score indicating the number of fulfilled metrics (e.g., 4 out of 7 for Findability and 14 out of 26 for the overall FAIRness). We extracted scores on January 22^nd^, 2026. All requests were processed by F-UJI and returned outputs. F-UJI automatically outputs FAIRness scores as percentages when retrieving data via the API (e.g., 50% FAIRness means that 50% of the total metrics were met), which we used for the subsequent analysis. Furthermore, while F-UJI automatically provides categorical FAIR levels (initial, moderate, and advanced) for each survey, these were not used in our study. The FAIRness scores obtained from F-UJI served as dependent variables in our analyses.

To obtain the FAIRness from F-UJI, we inputted a persistent identifier that linked to the survey data and their metadata (i.e., a DOI). If no DOI was available for a survey, we used the URL of the survey project website instead (if available). If DOI and URL were available, we used the DOI to ensure replicability of our approach. Among the 717 surveys collected in the dataset, not every one of them had a unique identifier. Some survey programs included multiple waves of data collection (i.e., surveys) and published these data using the same DOI. In these cases, we retrieved FAIRness scores for the identifier available to us, and consequently, all surveys received the same value. In total, we had 319 unique identifiers (N(DOI)=201 and N(URL)=118). We address this clustered data structure in our analytical strategy (see below).

As independent variables, we relied on measures of survey design characteristics, including sampling method and survey mode. Our operationalization followed the work by von Glasenapp et al. [7]. Regarding the sampling method, we differentiated between probability and non-probability sampling. Regarding survey mode, we differentiated between computer-assisted personal interview (CAPI), computer-assisted telephone interview (CATI), computer-assisted web interview (CAWI), paper-and-pencil interview (PAPI), and mixed modes (i.e., combinations of two or more modes). In addition, we created a variable combining the information on sampling and mode in five categories (0 = prob. CATI, 1 = prob. CAWI, 2 = prob. Mixed-Mode, 3 = prob. CAPI or PAPI, 4 = non-prob. CAWI, 5 = non-prob. Mixed-Mode).

### Analyses

In our analyses, we used data restricted to unique identifiers (N = 319). For this purpose, we excluded duplicate cases that shared a DOI or URL. For duplicates, we included only the earliest entry in the dataset. Fig 1 illustrates our exclusion process. Excluding duplicate cases was necessary as the SDCCP included multiple entries for longitudinal survey programs (see above) and would otherwise overrepresent these surveys.

**Fig 1:**
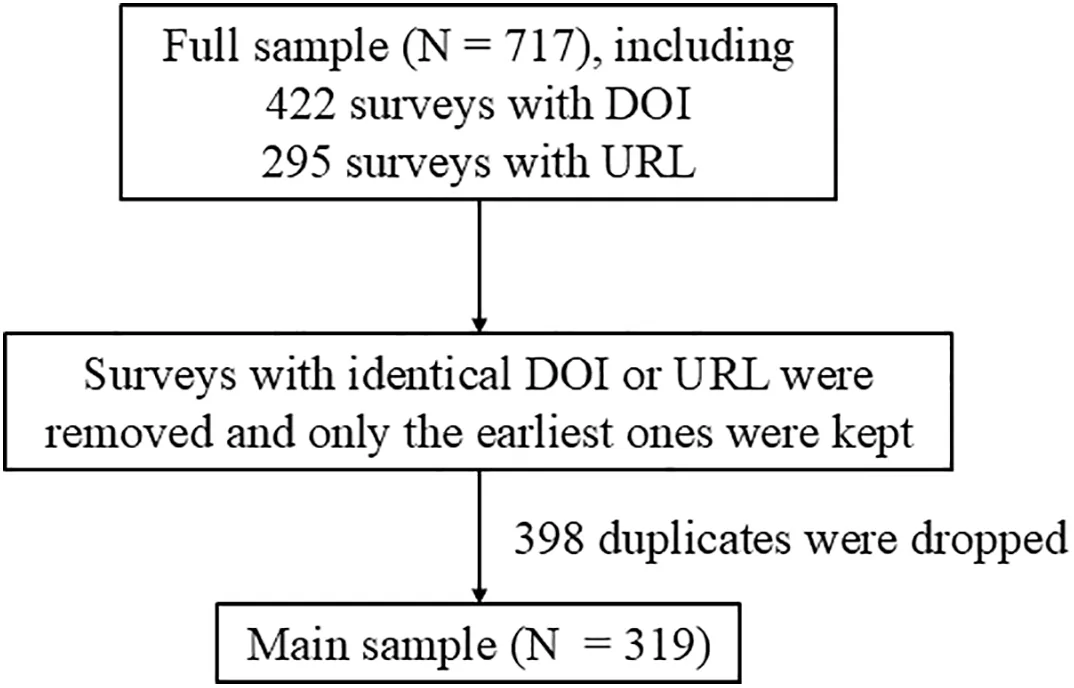
Flowchart of the Sample Selection.

To answer RQ1 (“How FAIR are survey data collected during the COVID-19 pandemic in Germany?”), we provided descriptive statistics of the FAIRness measures we obtained for the surveys. We complemented total FAIRness scores with FAIRness of subdimensions for a more comprehensive description.

To answer RQ2 (“Does the FAIRness of survey data collected during the COVID-19 pandemic in Germany differ by survey design?”), we used descriptive statistics conditional on sampling method and survey mode. In addition, we ran an Ordinary Least Squares (OLS) regression model with the overall FAIRness as the dependent variable and the combined survey design variable as the independent variable. For additional insights, we reran the model twice with the sampling method and mode as independent variables instead. We decided to use OLS due to its general robustness and easy accessibility of results to readers (for a robustness check of the model choice, see below).

*Robustness checks.* We performed a series of robustness checks. First, we investigated whether the existence of a DOI (instead of only providing a URL) affected FAIRness scores. We assumed that a persistent identifier was used when data were made available via data archives that routinely require researchers to provide a minimum of structured information about the survey. We compared FAIRness between those surveys that had a DOI and those that did not. Additionally, we fitted an OLS regression and estimated the effect of an existing DOI on FAIRness scores. To investigate whether DOI availability would impact our findings regarding RQ2, we re-ran our regression models for subsamples: those surveys with a DOI and those without a DOI. We would like to caution that these analyses should not be overinterpreted, in our view, as the availability of a DOI often indicates a decision by survey researchers to publish data via an archive or repository. These archives follow established practices and provide structured metadata that impact the FAIRness score. In this sense, our robustness check serves the purpose of clarifying how much impact this specific decision of data producers for archiving has on the FAIRness score.

Second, we reran our analyses for the full dataset without excluding duplicates. Here, the full sample (N = 717) was used for our analyses. We conducted these analyses for readers who would take the perspective that each survey (wave) should be considered equally, independent of whether it is part of a longitudinal survey program or not. To account for the clustered data structure of the full sample, we re-ran the regression model with cluster-robust standard errors. We further tested a multi-level model but obtained an Intra Class Correlation (ICC) of 0.966, indicating that between survey differences accounted for most of the variation. As including within survey variation would not improve prediction, we discarded this option.

Third, some survey design categories were sparsely populated (e.g., probability-based mixed-mode designs). To avoid overinterpreting unstable estimates, we reran our analyses with separate models for RQ2, including mode and sampling procedure as individual independent variables. To account for the fact that the full sample would yield more cases (which then, however, would be clustered, see above), we additionally reran these models with cluster-robust standard error for the full sample (N = 717).

Fourth, to answer RQ2, we used OLS regressions. As our dependent variables are bounded percentage outcomes, we conducted a robustness check in which we fitted a fractional logit model with overall FAIRness as the dependent variable and the combined survey design variable as the independent variable.

## Results

### RQ1

Fig 2 shows the distribution of FAIRness among surveys collected during the COVID-19 pandemic. The mean score among the survey was 18.97 out of a maximum of 26 (SD = 4.94), which amounts to 72.96% of FAIRness. The survey that performed the best received 100% of the score, while the worst one received 11.54%. What we observed from the distribution of FAIRness scores is that surveys tend to form two groups. One group of surveys achieved higher scores of around 88.46%, whereas the other achieved scores of about 65.38%.

**Fig 2:**
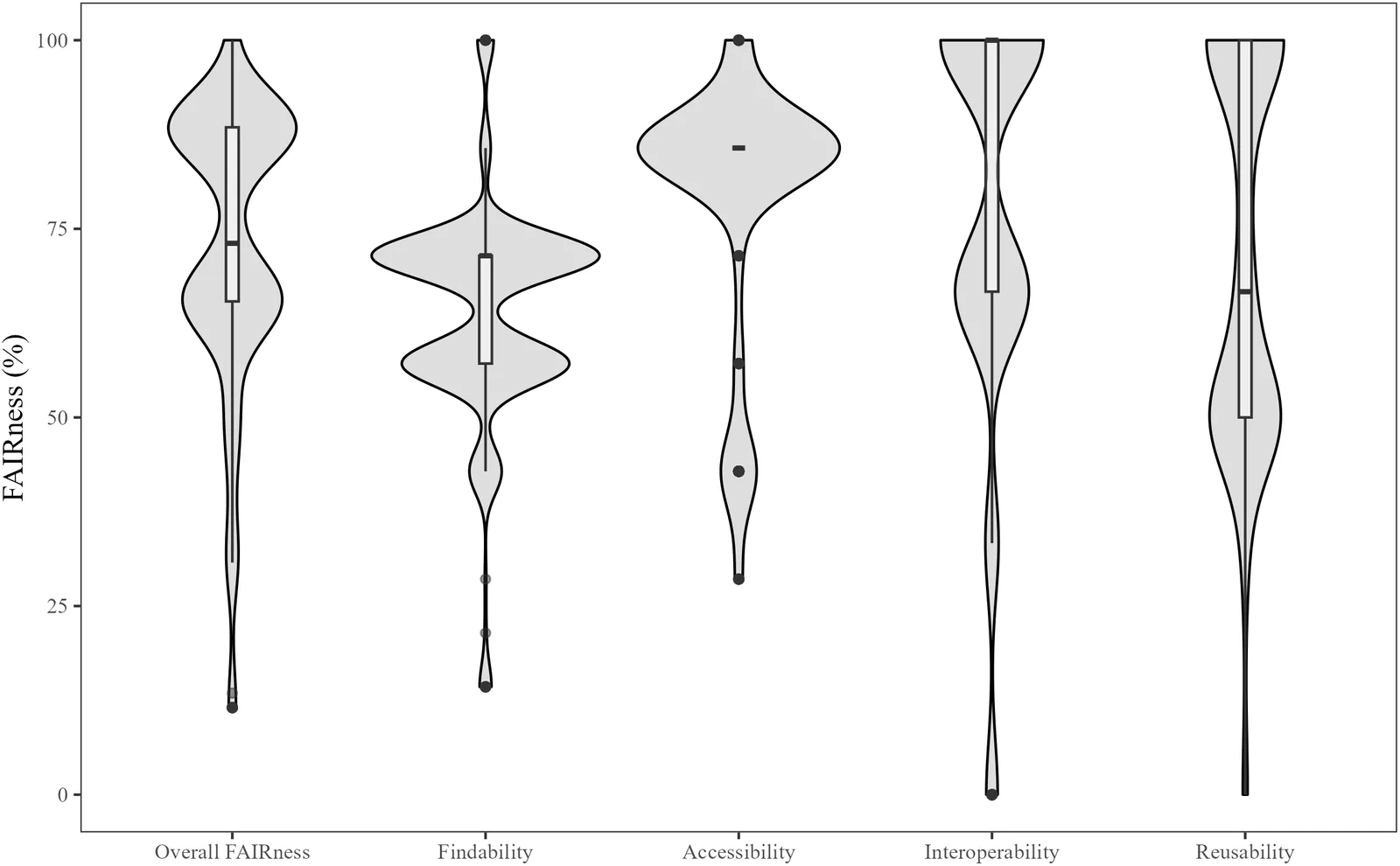
Distribution of FAIRness of Surveys During the COVID-19 Pandemic. **Note.** The figure includes a combination of boxplots with whiskers and outliers as well as onion plots that show the density of the distributions.

Looking into dimensions of the FAIRness revealed a more nuanced picture. We found that surveys performed best regarding Accessibility with 78.81% on average, followed by Interoperability at 77.74%, Reusability at 71.89%, and, finally, Findability averaging 63.93% of the total score. Again, for each dimension, we observed grouping that differed between dimensions: a majority of surveys were at 85.71% for Accessibility, whereas two groups each can be differentiated for Findability (at 57.14% and 71.43%), Interoperability (at 66.67% and 100.00%), and Reusability (50.00% and 100.00%).

Overall, FAIRness scores varied across the 319 surveys in our data. Here, not every survey received a FAIRness score we would deem desirable. When looking into variation between surveys, especially when focusing on subdimensions, we found grouping that we interpreted as a first indication of systematic differences between surveys. There seems to be a group of surveys that generally achieved a higher level of FAIRness, ensuring the availability and reusability of their data. On the other hand, there seems to be a group of surveys that is not as readily available and accessible for reuse by external researchers.

### RQ2

To investigate systematic differences between the FAIRness of surveys in our data, we analyzed FAIRness conditional on their survey design (see Table 1). The use of probability sampling and either CAPI or PAPI mode coincided with the highest average FAIRness of 82.69%, which is closely followed by surveys using probability sampling and CATI mode with FAIRness at 79.81%. The lowest scores in this regard were obtained by surveys using non-probability sampling, regardless of their survey mode.

**Table 1:** Summary statistics of FAIRness across different survey designs.

Design Groups	N	Mean	SD	Median	IQR	Min	Max
Prob./CATI	113	79.81	18.68	88.46	0.00	11.54	100.00
Prob./CAWI	24	73.08	24.51	88.46	24.04	11.54	88.46
Prob./mixed	18	73.40	24.13	88.46	31.73	13.46	100.00
Prob./CAPI or PAPI	12	82.69	12.33	88.46	1.92	50.00	88.46
Nonprob./CAWI	135	67.55	16.54	65.38	7.70	11.54	100.00
Nonprob./mixed	1	65.38		65.38	0.00	65.38	65.38

**Note.** N = 303, due to 16 missing values in the survey design variable.

Our OLS regression confirmed the previous findings (Table 2, Model 1). Our model showed the large differences between probability-based CATI surveys (reference category) and non-probability web surveys (β = −12.268), p <  .001).

**Table 2:** Regression results on the relationship between FAIRness scores and survey design, sampling method, and survey mode.

	Model 1	Model 2	Model 3
**Ref: Prob./CATI**			
Prob./CAWI	−6.739		
	(4.148)		
			
Prob./mixed	−6.419		
	(4.684)		
			
Prob./CAPI or PAPI	2.876		
	(5.603)		
			
Nonprob./CAWI	−12.268^***^		
	(2.353)		
			
Nonprob./mixed	−14.435		
	(18.537)		
			
**Ref: Probability**			
Non-Probability		−10.889^***^	
		(2.119)	
			
**Ref: CAPI**			
CATI			−6.081
			(6.297)
			
CAWI			−17.697^**^
			(6.220)
			
PAPI			−14.743
			(10.925)
			
Mixedmode			−12.921
			(7.357)
			
Constant	79.815^***^	78.361^***^	85.896^***^
	(1.736)	(1.425)	(6.060)
			

Observations	303	305	313
R^2^	0.093	0.080	0.094
Adjusted R^2^	0.078	0.077	0.083
Residual Std. Error	18.455 (df = 297)	18.417 (df = 303)	18.181 (df = 308)
F Statistic	6.092^***^ (df = 5; 297)	26.412^***^ (df = 1; 303)	8.029^***^ (df = 4; 308)

**Note.** Regression coefficients, standard errors in brackets. *p < .05; **p < .01; ***p < .001

Focusing only on the sampling method (Table 2, Model 2) adds nuance to the findings for the combined survey design variable. Surveys using probability sampling scored an average of 78.36% of FAIRness, which was approximately 10 percentage points higher than surveys using non-probability sampling (67.47%). Again, we found this pattern in our regression analyses (β = −10.889), p < .001; Table 2, Model 2).

Similarly, focusing only on survey mode (Table 2, Model 3), surveys that used CAPI obtained the highest FAIRness at 85.9%, whereas those using CAWI mode averaged the lowest at 68.2%. In our regression models (Table 2, Model 3), we observed the same effect (β = −17.697), p < .01).

Overall, our analysis shows an association between survey design and FAIRness of data. Those surveys that utilized designs aimed at fast and cost-efficient production of survey data were also less likely to invest in comprehensive and accessible documentation to make their data easy to reuse.

### Robustness checks

#### Existence of a DOI.

Those surveys where a DOI was available generally received better FAIRness scores compared to those for which no DOI was available and a website link had to be used (β = 23.875, p < .001, see S1 Table). On average, surveys with a DOI received a FAIRness score of 81.79%, whereas those without a DOI averaged at 57.92%.

Regarding RQ2, we found that probability-based mixedmode and non-probability CAWI surveys received lower FAIRness scores among those surveys with a DOI, compared to the reference category (see S2 Table). Among those without a DOI, we found non-probability CAWI to have higher FAIRness scores compared to the reference. These findings should be interpreted with caution as the existence of a DOI was unequally distributed across designs (see S3 Table): probability-based surveys mostly had a DOI (prob. CATI: 86.73%, prob. CAWI: 79.17%, prob. Mixed: 88.89%, prob. CAPI or PAPI: 91.67%), whereas nonprobability surveys did not (nonprob. CAWI: 40.74%, nonprob. Mixed: 0%). Sparse group sizes, especially among probability-based surveys without a DOI, are a likely explanation for the odd findings here. Having said that, these robustness checks show that higher availability of DOIs among probability-based surveys likely explains a share of the higher FAIRness scores of these designs compared to nonprobability surveys.

#### Inclusion of duplicates.

In general, our robustness checks are in line with our previous findings. Regarding RQ1, FAIRness amounted to 71.81% with duplicates (SD = 15.38, Min = 11.54%, Max = 100%). In terms of subdimensions, Accessibility still achieved the highest FAIRness at 78.26%, whereas Findability received the lowest average at 64.81%.

Regarding RQ2 (S4 Table, Model 1), as before, we found non-probability CAWI surveys to have lower FAIRness compared to the reference category. Considering the clustered data structure yields the same findings. Surprisingly, in this model, we also found non-probability mixed-mode surveys, a sparsely populated group, to show lower FAIRness compared to the reference as well. In general, these replications using the full sample are in line with the conclusion of our main model.

#### Small-N design groups.

Generally, we find patterns supporting our previous conclusions (Table 2, Models 2 and 3). Nonprobability designs are associated with lower FAIRness scores, whereas probability-based designs are associated with higher FAIRness scores. Similarly, CAWI designs were associated with lower FAIRness scores compared to CAPI. Similar findings were obtained even when considering the full sample in a regression model with cluster-robust standard errors (S4 Table, Models 2 and 3).

#### OLS model choice.

Our robustness check using a fractional logit model yielded findings in support of our OLS models (S5 Table): we would have drawn the same substantive conclusions even when using a fractional logit instead of the OLS model.

## Conclusion

We set out to investigate the FAIRness of survey data collected in Germany during the COVID-19 pandemic. During this crisis, survey data provided an important foundation to study the population’s attitudes, health-related behaviors, and opinions. Previous studies on data collection practice during the COVID-19 pandemic showed that researchers were facing a trade-off between quickly obtaining data and publishing results versus focusing on implementing high-quality, yet time-consuming, data collection procedures [7–9]. These studies showed that, as a result of this trade-off, surveys that focused on quick data collection and employed the necessary designs for thiswere also less likely to publish data quickly and with comprehensive data documentation. With the present study, we addressed research gaps in this regard. Our data revealed strong variation between the FAIRness scores of surveys (RQ1). We found them to differ systematically, with one group of surveys generally receiving higher scores at around 88.46% of FAIRness, whereas another group of surveys averaged at around 65.38%% FAIRness. This systematic pattern is related to the survey design used during data collection (RQ2). Surveys that employed designs that enabled quick and cost-efficient data collection obtained lower FAIRness scores compared to those with more costly and time-intensive survey designs. In this regard, our findings add further evidence to previous research on data collection practices during the COVID-19 pandemic [6–9] and that the trade-off of collecting data and producing findings quickly also extended to the Findability, Accessibility, Interoperability, and Reusability of these research data.

Our findings have implications for survey researchers, research data management, and data users in general. First, while the need for rapid data collection during crises such as the COVID-19 pandemic is understandable, the FAIRness of data should be considered. Research data are a common good, and enabling replication and transparency are necessary elements of the scientific process. The need to allow replication is especially important when taking on risk regarding the generalizability of findings when operating with survey designs that are at risk of producing lower data quality. Our findings are troublesome news in this regard; exactly those surveys that would require replication to ensure the robustness of their findings obtained the lowest FAIRness scores. Second, academic research is mostly publicly funded. Accordingly, it should be in the interest of funding agencies to make research data publicly available to ensure efficient use of the resources invested and robust results due to replication. As our robustness checks highlighted the importance of publishing data via repositories that require systematic metadata and provide persistent identifiers (i.e., DOIs), we recommend that funding agencies more strongly nudge researchers to publish their research data this way to facilitate the provision of proper metadata.

Our study surely is not without limitations, which future research could address. First, we focused on surveys that were included in the SDCCP data. The SDCCP project did not include every survey, especially not those that were not published anywhere or those that had so little documentation that they were discarded during eligibility testing. In this regard, our study systematically overestimates FAIRness. We therefore caution that real differences and FAIRness scores of surveys in the field will likely be lower.

Second, we relied on automated FAIRness assessment using F-UJI. While this decision enabled us to effectively process documentation for a large number of surveys, the automated procedure has limitations. F-UJI measures require detectable compliance with specific technical patterns and embedded structured metadata. These will be found more likely on DOI-referenced archive-published data compared to project websites. These might even contain rich study documentation, but the algorithm might fail to detect them due to unexpected information structure. For those surveys, for which we only had a URL, we found that FAIRness scores were lower. Future studies could investigate this issue further, for instance, by applying expert review of selected cases to assess possible biases. However, we would argue that the decision to make data available via a data archive or repository that ensures long-term availability as well as systematic and detectable metadata is a decision within the scope of FAIRness itself and, thus, should be considered during such analyses.

Third, we focused on surveys conducted in Germany during the COVID-19 pandemic. Germany is an example of an advanced research landscape with well-developed research infrastructures, for which general knowledge about the need for replicability and transparency in the field should be expected. Focusing on Germany allowed us to draw on an existing collection of metadata on surveys and keep the country context of each survey comparable. However, we would welcome replication in other countries or disciplines. We also see benefits in replicating our analyses without the focus on the COVID-19 pandemic. Analyzing more surveys, however, will require coding the relevant metadata to identify survey design characteristics. The survey selection and coding procedure will require additional resources and time. The procedures developed as part of the SDCCP project, from which we reused data, might serve as a blueprint for such research.

## Supporting information

S1 Table Regression results on the relationship between existence of a DOI and FAIRness scores. (DOC)

S2 Table Regression results on the relationship between survey design and FAIRness scores for surveys with a DOI and surveys without a DOI. (DOC)

S3 Table Proportion of surveys with or without a DOI by survey design (in %). (DOCX)

S4 Table Regression results on the relationship between survey design and FAIRness scores for the full sample. (DOC)

S5 Table Results from OLS Model and Fractional Logit Model. (DOC)

## References

[1] Pierce M, McManus S, Jessop C, John A, Hotopf M, Ford T, et al. Says who? The significance of sampling in mental health surveys during COVID-19. Lancet Psychiatry. 2020;7(7):567–8. doi: 10.1016/S2215-0366(20)30237-6 32502467 PMC7266586

[2] Eichenberger S, Varone F, Sciarini P, Stähli R, Proulx J. When do decision makers listen (less) to experts? The Swiss government’s implementation of scientific advice during the COVID‐19 crisis. Policy Studies Journal. 2023;51(3):587–605. doi: 10.1111/psj.12494

[3] Hadorn S, Sager F, Mavrot C, Malandrino A, Ege J. Evidence-based policymaking in times of acute crisis: Comparing the use of scientific knowledge in Germany, Switzerland, and Italy. Polit Vierteljahresschr. 2022;63(2):359–82. doi: 10.1007/s11615-022-00382-x 35399336 PMC8977835

[4] Jung RG, Di Santo P, Clifford C, Prosperi-Porta G, Skanes S, Hung A, et al. Methodological quality of COVID-19 clinical research. Nat Commun. 2021;12(1):943. doi: 10.1038/s41467-021-21220-5 33574258 PMC7878793

[5] Nieto I, Navas JF, Vázquez C. The quality of research on mental health related to the COVID-19 pandemic: A note of caution after a systematic review. Brain Behav Immun Health. 2020;7:100123. doi: 10.1016/j.bbih.2020.100123 32835299 PMC7395946

[6] von Glasenapp K. Evaluating the accuracy of survey data: A case study of COVID-19 vaccination rates in Germany. BMC Med Res Methodol. 2025;25(1):234. doi: 10.1186/s12874-025-02702-2 41126070 PMC12541969

[7] von Glasenapp K, Skora T, Gummer T. Survey design and quality during the COVID-19 pandemic in Germany: An assessment with 686 social science surveys. Surv Res Methods. 2026. doi: 10.18148/srm/2026.v20i1.8447

[8] Gummer T, von Glasenapp K, Skora T, Bartholomäus S, Naumann E. The impact of the COVID-19 pandemic on the design of repeated cross-sectional and panel surveys in Germany. Int J Public Opinion Res. 2025;37(4). doi: 10.1093/ijpor/edaf062

[9] Gummer T, von Glasenapp K, Skora T, Naumann E, Bartholomäus S, Piesch S. Survey data collection during the COVID-19 pandemic in Germany: Recommendations for an improved data collection infrastructure. Health Policy. 2026;165:105551. doi: 10.1016/j.healthpol.2025.105551 41447855

[10] Jacobsen A, De Miranda Azevedo R, Juty N, Batista D, Coles S, Cornet R. FAIR principles: Interpretations and implementation considerations. Data Intell. 2020;2:10–29. doi: 10.1162/dint_r_00024

[11] Mons B, Neylon C, Velterop J, Dumontier M, da Silva Santos LOB, Wilkinson MD. Cloudy, increasingly FAIR; revisiting the FAIR Data guiding principles for the European open science cloud. Information Services and Use. 2017;37(1):49–56. doi: 10.3233/isu-170824

[12] Wilkinson MD, Dumontier M, Aalbersberg IJJ, Appleton G, Axton M, Baak A, et al. The FAIR guiding principles for scientific data management and stewardship. Sci Data. 2016;3:160018. doi: 10.1038/sdata.2016.18 26978244 PMC4792175

[13] Eder C, Jedinger A. FAIR national election studies: How well are we doing?. Eur Polit Sci. 2019;18:651–68. doi: 10.1057/s41304-018-0194-3

[14] Zenk-Möltgen W. The role of FAIR principles in high-quality research data documentation: Looking at national election studies. IQ. 2025;49(2). doi: 10.29173/iq1119

[15] Batzdorfer V, Zenk-Möltgen W, Young L, Katsanidou A, Breuer J, Bishop L. Between urgency and data quality: assessing the FAIRness of data in social science research on the COVID-19 pandemic. Res Ethics. 2024;20(4):744–63. doi: 10.1177/17470161241257575

[16] Sofi-Mahmudi A, Raittio E, Khazaei Y, Ashraf J, Schwendicke F, Uribe SE, et al. COVID-19-related research data availability and quality according to the FAIR principles: A meta-research study. PLoS One. 2024;19(11):e0313991. doi: 10.1371/journal.pone.0313991 39556553 PMC11573139

[17] Jabkowski P. Increase in the quality of methodological documentation of cross-national pan-European multi-wave surveys over the last 40 years – a research note. Int J Soc Res Methodol. 2023;26:817–24. doi: 10.1080/13645579.2022.2097394

[18] Kołczyńska M, Schoene M. Survey Data Harmonization and the Quality of Data Documentation in Cross‐national Surveys. Advances in Comparative Survey Methods. Wiley. 2018. 963–84. doi: 10.1002/9781118884997.ch44

[19] Stefkovics Á, Eichhorst A, Skinnion D, Harrison CH. Are we becoming more transparent? Survey reporting trends in top journals of social sciences. Int J Public Opin Res. 2024;36:edae013. doi: 10.1093/ijpor/edae013

[20] Gummer T, Skora T, von Glasenapp K, Naumann E. A dataset on survey designs and quality of social and behavioral science surveys during the COVID-19 pandemic. Sci Data. 2024;11(1):619. doi: 10.1038/s41597-024-03475-x 38866799 PMC11169371

[21] von Glasenapp K, Skora T, Gummer T, Naumann E. SDCCP 1 - Survey Design and Quality During the Covid-19 Pandemic. Cologne: GESIS Data Archive. 2024. doi: 10.7802/2652 PMC11169371 38866799

[22] Devaraju A, Huber R. An automated solution for measuring the progress toward FAIR research data. Patterns (N Y). 2021;2(11):100370. doi: 10.1016/j.patter.2021.100370 34820651 PMC8600246

[23] Devaraju A, Huber R. F-UJI - An Automated FAIR Data Assessment Tool. Zenodo. 2025. doi: 10.5281/zenodo.17350027

