# Supplementary material for: Assessing the FAIRness of survey data collected during the COVID-19 pandemic in Germany
Source: PLoS One. 2026 Oct 9;21(10):e0358755. doi: 10.1371/journal.pone.0358755 (PMC13652268; doi:10.1371/journal.pone.0358755)
Supplement: Supplementary file 1 [file pone.0358755.s003.docx]

| Survey Design | Without DOI | With DOI |
| --- | --- | --- |
| Prob./CATI | 13.27% | 86.73% |
| Prob./CAWI | 20.83% | 79.17% |
| Prob./mixed | 11.11% | 88.89% |
| Prob./CAPI or PAPI | 8.33% | 91.67% |
| Nonprob./CAWI | 59.26% | 40.74% |
| Nonprob./mixed | 100% | 0% |
