# Supplementary material for: Assessing the FAIRness of survey data collected during the COVID-19 pandemic in Germany
Source: PLoS One. 2026 Oct 9;21(10):e0358755. doi: 10.1371/journal.pone.0358755 (PMC13652268; doi:10.1371/journal.pone.0358755)
Supplement: Supplementary file 2 [file pone.0358755.s001.doc]

|  |  |
| --- | --- |
|  | |
|  | Average FAIRness |
|  | |
| DOI Exists | 23.875^\*\*\*^ |
|  | (1.750) |
|  |  |
| Constant | 57.917^\*\*\*^ |
|  | (1.389) |
|  |  |
|  | |
| Observations | 319 |
| R^2^ | 0.370 |
| Adjusted R^2^ | 0.368 |
| Residual Std. Error | 15.092 (df = 317) |
| F Statistic | 186.066^\*\*\*^ (df = 1; 317) |
|  | |
|  | |

**Note.** Regression coefficients, standard
errors in brackets. \*p<.05; \*\*p<.01; \*\*\*p<.001
