# Supplementary material for: Assessing the FAIRness of survey data collected during the COVID-19 pandemic in Germany
Source: PLoS One. 2026 Oct 9;21(10):e0358755. doi: 10.1371/journal.pone.0358755 (PMC13652268; doi:10.1371/journal.pone.0358755)
Supplement: Supplementary file 3 [file pone.0358755.s002.doc]

|  |  |  |
| --- | --- | --- |
|  | | |
|  | Surveys with DOI | Surveys without DOI |
| **Ref: Prob./CATI** |  |  |
| Prob./CAWI | -2.848 | -6.153 |
|  | (3.475) | (7.825) |
|  |  |  |
| Prob./mixed | -11.040^\*\*^ | 22.305 |
|  | (3.738) | (11.407) |
|  |  |  |
| Prob./CAPI or PAPI | -1.174 | 22.305 |
|  | (4.409) | (15.650) |
|  |  |  |
| Nonprob./CAWI | -7.887^\*\*\*^ | 17.594^\*\*\*^ |
|  | (2.336) | (4.263) |
|  |  |  |
| Nonprob./mixed |  | 22.305 |
|  |  | (15.650) |
|  |  |  |
| Constant | 85.438^\*\*\*^ | 43.075^\*\*\*^ |
|  | (1.401) | (3.912) |
|  |  |  |
|  | | |
| Observations | 199 | 104 |
| R^2^ | 0.080 | 0.218 |
| Adjusted R^2^ | 0.061 | 0.178 |
| Residual Std. Error | 13.865 (df = 194) | 15.153 (df = 98) |
| F Statistic | 4.206^\*\*^ (df = 4; 194) | 5.469^\*\*\*^ (df = 5; 98) |
|  | | |
|  | | |

**Note.** Regression coefficients, standard errors in
brackets. \*p<.05; \*\*p<.01; \*\*\*p<.001
