# Supplementary material for: Assessing the FAIRness of survey data collected during the COVID-19 pandemic in Germany
Source: PLoS One. 2026 Oct 9;21(10):e0358755. doi: 10.1371/journal.pone.0358755 (PMC13652268; doi:10.1371/journal.pone.0358755)
Supplement: Supplementary file 4 [file pone.0358755.s004.doc]

|  |  |  |  |
| --- | --- | --- | --- |
|  | | | |
|  | Model 1 | Model 2 | Model 3 |
| **Ref: Prob./CATI** |  |  |  |
| Prob./CAWI | 3.178 |  |  |
|  | (4.512) |  |  |
|  |  |  |  |
| Prob./mixed | 1.049 |  |  |
|  | (5.742) |  |  |
|  |  |  |  |
| Prob./CAPI or PAPI | 4.731 |  |  |
|  | (4.680) |  |  |
|  |  |  |  |
| Nonprob./CAWI | -9.897^\*\*\*^ |  |  |
|  | (2.836) |  |  |
|  |  |  |  |
| Nonprob./mixed | -11.249^\*\*\*^ |  |  |
|  | (2.683) |  |  |
|  |  |  |  |
| **Ref: Probability** |  |  |  |
| Non-Probability |  | -10.610^\*\*\*^ |  |
|  |  | (2.361) |  |
|  |  |  |  |
| **Ref: CAPI** |  |  |  |
| CATI |  |  | -9.267^\*^ |
|  |  |  | (3.617) |
|  |  |  |  |
| CAWI |  |  | -17.498^\*\*\*^ |
|  |  |  | (2.706) |
|  |  |  |  |
| PAPI |  |  | -15.898^\*^ |
|  |  |  | (6.627) |
|  |  |  |  |
| Mixed-mode |  |  | -9.408 |
|  |  |  | (5.485) |
|  |  |  |  |
| Constant | 76.629^\*\*\*^ | 77.348^\*\*\*^ | 85.896^\*\*\*^ |
|  | (2.683) | (2.189) | (2.429) |
|  |  |  |  |
|  | | | |
| Observations | 691 | 695 | 704 |
| R^2^ | 0.124 | 0.120 | 0.080 |
| Adjusted R^2^ | 0.117 | 0.119 | 0.075 |
| Residual Std. Error | 14.444 (df = 685) | 14.395 (df = 693) | 14.686 (df = 699) |
| F Statistic | 19.329^\*\*\*^ (df = 5; 685) | 94.333^\*\*\*^ (df = 1; 693) | 15.243^\*\*\*^ (df = 4; 699) |
|  | | | |
|  | | | |

**Note.** Regression coefficients, cluster robust standard errors in brackets.
\*p<.05; \*\*p<.01; \*\*\*p<.001
