# Supplementary material for: Assessing the FAIRness of survey data collected during the COVID-19 pandemic in Germany
Source: PLoS One. 2026 Oct 9;21(10):e0358755. doi: 10.1371/journal.pone.0358755 (PMC13652268; doi:10.1371/journal.pone.0358755)
Supplement: Supplementary file 5 [file pone.0358755.s005.doc]

|  |  |
| --- | --- |
|  | |
|  | Fractional Logit Model |
|  | |
| Prob./CAWI | -0.376 |
|  | (0.221) |
|  |  |
| Prob./mixed | -0.360 |
|  | (0.249) |
|  |  |
| Prob./CAPI or PAPI | 0.189 |
|  | (0.341) |
|  |  |
| Nonprob./CAWI | -0.642^\*\*\*^ |
|  | (0.127) |
|  |  |
| Nonprob./mixed | -0.739 |
|  | (0.904) |
|  |  |
| Constant | 1.375^\*\*\*^ |
|  | (0.100) |
|  |  |
|  | |
| Observations | 303 |
|  | |
|  | |

**Note.** Regression coefficients, standard errors in
brackets. \*p<.05; \*\*p<.01; \*\*\*p<.001
